# Diet quality, food intake and incident adult-onset asthma: a Lifelines Cohort Study

**DOI:** 10.1007/s00394-023-03091-2

**Published:** 2023-02-04

**Authors:** Edith Visser, Kim de Jong, Janneke J. S. Pepels, Huib A. M. Kerstjens, Anneke ten Brinke, Tim van Zutphen

**Affiliations:** 1 Department of Epidemiology, Medical Centre Leeuwarden, Leeuwarden, The Netherlands; 2 Department of Sustainable Health, Faculty Campus Fryslân, University of Groningen, Leeuwarden, The Netherlands; 3 Department of Pulmonary Medicine, University of Groningen, University Medical Centre Groningen, Groningen, The Netherlands; 4 Department of Pulmonary Medicine, Medical Centre Leeuwarden, Leeuwarden, The Netherlands

**Keywords:** Adult-onset asthma, Nutrition, Food groups, Obesity, Incidence, Epidemiology

## Abstract

**Purpose:**

Dietary factors have been suggested as drivers of the rising prevalence of adult-onset asthma, but evidence is inconclusive, possibly due to the complex interrelation with obesity. We aim to explore the relation of diet quality and food intake with incident adult-onset asthma in normal weight and overweight adults of the prospective population-based Lifelines Cohort Study.

**Methods:**

Incident adult-onset asthma was defined as self-reported asthma at ± 4-year follow-up, in adults free of airway disease at baseline. Diet quality scores and food group intake were assessed at baseline. Log-binomial regression analyses were used to estimate adjusted relative risks (RR) between dietary intake (per portion) and incident adult-onset asthma, in categories of BMI (cutoff: 25 kg/m^2^).

**Results:**

477 incident asthma cases (75% female, 62% overweight) and 34,698 controls (60% female, 53% overweight) were identified. Diet quality—assessed by the Lifelines Diet Score and Mediterranean Diet Score—was not associated with incident adult-onset asthma in the two BMI groups. Although the dietary intake of several food groups differed between cases and controls, after adjustment for confounders only few remained associated with adult-onset asthma, including red and processed meat (RR: 0.93 per 15 g intake; 95% CI 0.86–0.99) in the normal weight group and intake of cheese (RR 1.09 per 20 g intake; 95% CI 1.00–1.17) and vegetables (RR 1.10 per 50 g intake; 95% CI 1.00–1.21) in the overweight group.

**Conclusion:**

The results of this study question the role of food as a ‘simple’ predictor of adult-onset asthma and call for an integrative approach, including a range of modifiable lifestyle factors and further asthma phenotyping.

**Supplementary Information:**

The online version contains supplementary material available at 10.1007/s00394-023-03091-2.

## Introduction

Asthma is a complex and heterogeneous airway disease, characterized by airway inflammation and bronchial hyperresponsiveness, resulting in recurrent episodes of dyspnea, wheezing and cough. Asthma is typically known as a disease that begins in early childhood and is associated with atopy and familial heredity. However, other forms of asthma have been recognized since the mid-twentieth century, and the clinical relevance of asthma starting in adulthood has since received more attention [1, 2]. Adult-onset asthma is often a severe and uncontrolled form and poses a large burden on both patients and healthcare, due to frequent hospitalizations and high oral corticosteroid use [1, 3]. Despite its burden, it remains largely unknown why asthma develops in adults [4].

The incidence of adult-onset asthma over the past decades is stable, with an incidence rate ranging from 3.6 to 4.8 per 1000 person-years [5–7]. While asthma in childhood is often associated with exposure to specific allergens such as house dust mite, animal dander and pollen, a different etiology has been proposed in adult-onset asthma including environmental and lifestyle factors. Several dietary hypotheses have been put forward, for example the shift to a more pro-inflammatory and energy-rich diet [8–10]. This so-called ‘Western diet’ features high intake of animal products, saturated fat and refined sugars, while the nutritional value is relatively low in terms of antioxidants, omega-3 fatty acids and dietary fiber [9]. In contrast, the more plant-based Mediterranean diet is believed to have anti-inflammatory properties, largely attributed to the amount of fruit, vegetables, grains and nuts that are consumed [10]. These dietary factors can modulate epigenetics, gut microbiome composition, airway remodeling, innate immunity and inflammatory responses—all factors that have been implicated in the etiology of asthma [11, 12].

Thus far, prospective studies focusing on dietary factors and incident adult-onset asthma report contradictory results [13–29]. Yet, studies are very heterogeneous regarding the level of diet estimation—i.e., dietary patterns, diet scores, foods, or nutrients—and the definitions used for the assessment of asthma, making comparison of study findings a challenge [30, 31].

Furthermore, the Western diet has also been pinpointed as one of the main causes of the global obesity epidemic [31, 32]. Obesity itself is, however, considered an independent risk factor for adult-onset asthma [30, 32] and a potential effect modifier in the relation between diet and asthma [13]. This further complicates research into diet as risk factor of adult-onset asthma. To account for these complex interrelations, we aim to explore the relation of diet quality and food intake with incident adult-onset asthma at five year follow-up separately in normal weight and overweight adults from a general population.

## Methods

### Study design

This longitudinal study was performed using data of the Dutch Lifelines Cohort Study. Lifelines is a multi-disciplinary prospective population-based cohort study examining in a unique three-generation design the health and health-related behaviors of 152,638 persons ≥ 18 years living in the North of the Netherlands [33, 34]. It employs a broad range of investigative procedures in assessing the biomedical, socio-demographic, behavioral, physical and psychological factors which contribute to the health and disease of the general population, with a special focus on multi-morbidity and complex genetics. Participants were included in the cohort between 2006 and 2013 and written informed consent was obtained. At baseline and at the first follow-up assessment planned after approximately 5 years, participants were invited for a comprehensive health assessment including questionnaires, standardized anthropometric measurements, the collection of blood samples and spirometry following international criteria [35]. In addition, postal questionnaires were completed at 1.5 and 3 years after baseline, including a question about incident asthma. The Lifelines Cohort Study is being conducted according to the principles of the Declaration of Helsinki and approved by the Medical Ethics Committee of the University Medical Centre Groningen, the Netherlands.

### Assessment of incident adult-onset asthma

**Fig. 1:**
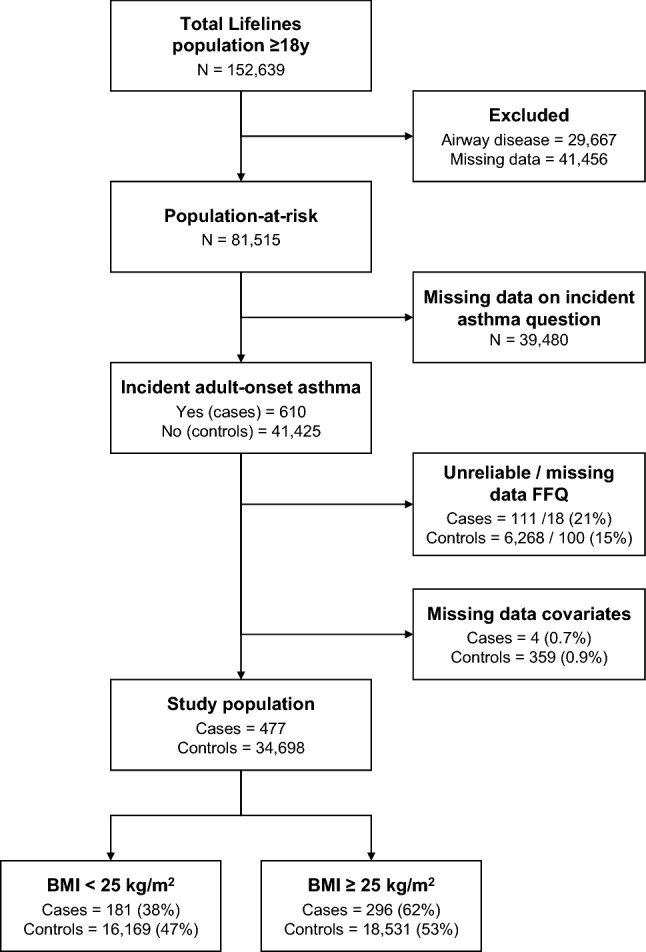
Flowchart of the included study population. BMI, body mass index; FFQ, food-frequency questionnaire

First, the population-at-risk was defined as all participants ≥ 18 years without chronic airway disease at baseline (Fig. 1). Chronic airway disease was determined by having either self-reported asthma, self-reported chronic obstructive pulmonary disease, airway obstruction according to spirometry values or respiratory medication use. Participants who fulfilled any of these criteria or who had missing data on any of these criteria were excluded. Second, adult-onset asthma at follow-up was assessed by the question “Have you developed asthma since the last time you filled in the Lifelines questionnaire”. This question was asked three times during our follow-up period, namely, at approximately 1.5, 3 and 5 years from baseline. Participants of the population-at-risk were considered a case of incident adult-onset asthma if they answered ‘yes’ at least once during follow-up. The control group consisted of the participants without any confirmative answer to this question (i.e., ‘yes’) during follow-up.

Cases and controls were split into normal weight (BMI < 25 kg/m^2^) and overweight (BMI ≥ 25 kg/m^2^) groups, according to WHO standards [36]. Body weight was measured without shoes by well-trained staff to the nearest 0.1 kg and height to the nearest 0.5 cm. Body mass index (BMI) was calculated as weight (kg) divided by height squared (m^2^).

### Dietary assessment

Dietary intake was assessed at baseline using a validated 110-item semi-quantitative food frequency questionnaire (FFQ) measuring food intake over the previous month [37]. For each item, participants reported the amount and frequency of consumption on a seven-point categorical scale—ranging from “not this month” to “6–7 days/week”—and estimated their portion size. Energy intake (kcal/day) was estimated from the FFQ data by using the 2011 Dutch food composition database [38]. Dietary intake data were considered unreliable when the ratio between reported energy intake and basal metabolic rate was below 0.50 or above 2.75, according to the Goldberg cutoff method [39, 40].

For analysis, FFQ data were categorized into 22 food groups and labeled as having positive, negative, or neutral/unknown health effects based on international evidence for diet–disease relations and the 2015 Dutch Dietary Guidelines [41, 42]. For this study, the consumption of food groups was expressed in grams/day using natural portion sizes and commonly used household measures (e.g., serving spoons) [43]. For the groups fish, red and processed meat and white unprocessed meat, a portion size of 100 g was divided by seven. This portion size equals an increase of one slice of cold cuts per day or one additional serving of meat/fish per week.

The Lifelines Diet Score (LLDS) was used as a measure of relative diet quality, ranging from 0 to 48 with higher values indicating a healthier diet [42]. This score consists of the nine positive food groups (vegetables, fruit, whole grain products, legumes and nuts, fish, oils and soft margarines, unsweetened dairy, coffee, tea) and three negative food groups (red and processed meat, butter and hard margarines, sugar-sweetened beverages).

The well-established Mediterranean Diet Score (MDS) was used to measure adherence to a Mediterranean-style diet [44]. The MDS is based on the consumption of nine selected food-items with the sex-specific median as cutoff. The components of the MDS are vegetables, legumes, fruit and nuts, cereals and fish as positive groups; meat, poultry and dairy as negative groups; and moderate alcohol intake. The MDS ranges from 0 to 9 points, with higher values indicating better adherence to a Mediterranean-style diet.

### Covariates assessment

Information on demographics (sex, age, educational level) and lifestyle (alcohol use, smoking, physical activity) was collected from the baseline questionnaires. Educational level was classified as low (no or primary education, lower secondary education, and lower vocational education), middle (higher secondary education and secondary vocational education) and high (higher vocational education and university education). Alcohol use in the previous month was measured and categorized into never, low (≤ once per week) and high (> once per week). Smoking status was categorized into never, former and current smoker. The lifetime number of pack-years smoked was calculated as the number of cigarette packs smoked per day (1 pack = 20 cigarettes) times the number of years of smoking. Physical activity was estimated as minutes per week of moderate-to-vigorous physical activity (MVPA) based on the Short Questionnaire to Assess Health-enhancing Physical Activity score (SQUASH) [45, 46]. Follow-up time was defined as the number of months between baseline and the first comprehensive assessment at approximately 5 years.

### Statistical analysis

Demographic and health-related characteristics are presented for cases and controls and stratified for normal weight (BMI < 25 kg/m^2^) and overweight (BMI ≥ 25 kg/m^2^). Results are given as mean ± SD (parametric data), median and interquartile range (non-parametric data), and percentages (categorical data). Between-group differences were tested accordingly with an independent sample *t* test, Mann–Whitney *U* test or Chi-square test.

The dietary intake of food groups were summarized using medians and IQR because the majority of variables had a non-parametric distribution. Comparisons between cases and controls were made with the Mann–Whitney *U* test in two categories of BMI (normal weight and overweight).

Multivariable log-binomial regression analyses were performed to estimate the relative risk (RR) and corresponding 95% CI for each food group and for the dietary scores LLDS and MDS on incident adult-onset asthma. The analyses were performed in the categories of BMI and adjusted for the covariates sex, age, educational level and follow-up time.

Sensitivity analyses were performed to examine the robustness of results. First, the multivariable regression analyses were repeated with inclusion of alcohol use, smoking exposure (log-transformed), moderate-to-vigorous physical activity (log-transformed) and energy intake. Second, in an additional regression analysis we also adjusted for food allergy, as food allergy is more common among asthma cases and might affect dietary intake. Third, given the complex relationship between diet, obesity and asthma, the models were re-run with inclusion of BMI as covariate to assess additional confounding by BMI. Last, a regression model was performed containing all food groups in addition to the standard covariates, to account for overall eating pattern.

The analyses were performed using IBM SPSS Statistics, version 25.0 (IBM, NY, USA). A *p* value < 0.05 was considered statistically significant.

## Results

### Population description

Among the population-at-risk (*n* = 81,515) who were free of airway disease at baseline, 610 cases of incident adult-onset asthma and 41,425 controls were identified at follow-up (Fig. 1). The population-at-risk was demographically similar to the overall population of the Lifelines Cohort Study (see online supplement, table S1). The mean ± SD follow-up time was 47 ± 11 months. After excluding participants with unreliable/missing FFQ data or missing covariate data, 477 cases remained in the analysis. Of these cases, 181 (38%) had a BMI < 25 kg/m^2^ and 296 (62%) a BMI ≥ 25 kg/m^2^. The control group consisted of 34,698 participants, of whom 18,531 (53%) were overweight.

The demographic and health-related characteristics of participants are described in Table 1. In both BMI groups, asthma cases were more frequently female and of younger age than controls. Furthermore, asthma cases had a lower alcohol use, but smoking behavior and physical activity were similar between the groups. In the overweight group, cases had a lower educational level, a higher BMI, and a lower energy intake than controls.

**Table 1:** Baseline characteristics of incident asthma cases and controls in categories of BMI

		BMI < 25 kg/m^2^		*p*	BMI ≥ 25 kg/m^2^		*p*
		Cases *N* = 181	Controls *N* = 16,169		Cases *N* = 296	Controls *N* = 18,531	
Follow-up time	Months	49 ± 11	47 ± 11	< 0.01	48 ± 11	46 ± 11	< 0.01
Sex	% Female	78.5	67.9	< 0.01	73.6	53.1	< 0.01
Age	Years	40.4 ± 13.3	44.3 ± 12.6	< 0.01	46.8 ± 12.5	49.1 ± 12.0	< 0.01
Educational level	% Low	18.8	22.3	0.50	34.1	33.8	< 0.01
	% Middle	41.4	38.6		46.6	38.6	
	% High	39.8	39.0		19.3	27.6	
BMI	kg/m^2^	22.5 ± 1.8	22.6 ± 1.7	0.40	29.7 ± 4.1	28.6 ± 3.3	< 0.01
MVPA	Min/week	270 (20–710)	295 (124–600)	0.91	240 (120–540)	270 (110–600)	0.41
Smoking status	% Current	17.2	16.5	0.94	17.3	15.6	0.71
	% Former	28.9	30.0		39.5	40.4	
	% Never	53.9	53.5		43.2	44.0	
Smoking exposure^a^	Pack-Years	5.6 (2.5–13.0)	6.5 (2.5–12.6)	0.80	9.0 (3.3–17.3)	9.1 (4.1–17.0)	0.73
Alcohol use	% Never	12.2	15.0	0.05	20.1	17.6	0.04
	% Low	43.3	34.7		38.4	33.3	
	% High	44.4	50.2		41.5	49.0	
Energy intake	kcal/day	2029 ± 673	2021 ± 578	0.40	1969 ± 563	2053 ± 601	0.02
Food allergy	% Yes	9.9	3.8	< 0.01	8,1	3,1	< 0.01
Nasal allergy	% Yes	58.0	26.8	< 0.01	41.5	24.7	< 0.01
FEV_1_	% Predicted	99.7 ± 12.0	105.7 ± 12.2	< 0.01	100.1 ± 13.9	105.3 ± 14.0	< 0.01
Eosinophils	Cells × 10^9^/L	0.18 (0.11–0.27)	0.14 (0.09–0.21)	< 0.01	0.18 (0.11–0.27)	0.15 (0.10–0.22)	< 0.01

Values are presented as %, mean ± SD or median (IQR)

^a^Deviant sample size, in former and current smokers only; *N* = 82/7119 for cases/controls with BMI < 25 kg/m^2^; *N* = 159/9811 for cases/controls with BMI ≥ 25 kg/m^2^. BMI, body mass index; FEV_1_, forced expiratory volume in 1 s; MVPA, moderate-to-vigorous physical activity

### Diet quality and the Mediterranean diet score

The Lifelines Diet Score ranged from 4 to 45 in the whole study population. In participants with a normal weight, asthma cases had a mean ± SD LLDS of 24.6 ± 6.0 and controls 24.7 ± 6.1 (*p* = 0.68). Similar results were observed in the overweight group with a mean LLDS of 24.7 ± 5.7 in asthma cases and 24.6 ± 5.9 in controls (*p* = 0.60). Also, after adjustment for confounders, the log-binomial regression analyses showed that diet quality—assessed by the LLDS—was not associated with incident adult-onset asthma in the normal weight group (RR 1.01, 95% CI 0.98–1.03) or in the overweight group (RR 1.01, 95% CI 0.99–1.03).

The Mediterranean Diet Score, ranging from 0 to 9, was comparable between cases and controls in both the normal weight (4.52 ± 1.54 in cases vs 4.40 ± 1.60 in controls, *p* = 0.28) and overweight group (4.19 ± 1.56 in cases vs 4.19 ± 1.56 in controls, *p* = 0.99). Better adherence to a Mediterranean-style diet was also not related to incident asthma in normal weight adults (RR 1.09, 95% CI 0.99–1.19) or overweight adults (RR 1.02, 95% CI 0.95–1.10) after adjustment for confounders.

### Dietary intake of food groups

The consumption of food groups differed between cases and controls, and between the two BMI groups (Table 2). Compared to controls, cases in the normal weight group had statistically significant lower intake of whole grains (median 94.2 vs 125.6 g/day, *p* = 0.04), unsweetened dairy (136.7 vs 150.1 g/day *p* = 0.02), coffee (348.4 vs 348.3 g/day, *p* < 0.01), soup (22.3 vs 35.8 g/day, *p* = 0.04), red and processed meat (58.1 vs 61.8 g/day, *p* = 0.03) and butter and hard margarines (16.1 vs 19.2 g/day, *p* = 0.03). The intake of tea (241.1 *vs* 232.2 g/day, *p* = 0.04) and sugar-sweetened beverages (120.1 *vs* 88.8 g/day, *p* = 0.03) was higher in asthma cases with a normal weight than controls.

**Table 2:** Dietary intake of 22 food groups in asthma cases and controls, in categories of BMI

	BMI < 25 kg/m^2^		*p*	BMI ≥ 25 kg/m^2^		*p*
	Cases *N* = 181	Controls *N* = 16,169		Cases *N* = 296	Controls *N* = 18,531	
Vegetables	108.7 (74.5–151.2)	108.9 (74.3–149.9)	0.99	109.8 (74.5–150.8)	108.3 (73.7–147.4)	0.05
Fruit	126.9 (42.3–220.2)	110.1 (76.2–220.2)	0.45	152.4 (76.2–220.2)	110.1 (42.3–220.2)	0.36
Whole grains	94.2 (62.8–125.6)	125.6 (65.2–157.0)	0.04	94.2 (62.8–125.6)	125.6 (65.2–157.0)	0.08
Legumes and nuts	16.4 (7.3–28,8)	17.1 (7.9–30.6)	0.25	17.6 (8.4–29.1)	16.9 (7.6–30.2)	0.79
Fish	10.9 (4.5–17.4)	10.7 (4.4–16.9)	0.63	10.2 (4.4–16.1)	10.9 (4.7–16.9)	0.07
Oils and soft margarines	14.1 (4.3–27.9)	13.9 (3.7–28.0)	0.87	13.0 (4.4–26.1)	14.9 (4.3–29.1)	0.15
Unsweetened dairy	136.7 (37.2–259.0)	150.1 (60.2–278.7)	0.02	151.9 (54.8–278.7)	155.6 (66.9–281.3)	0.54
Coffee	348.4 (80.4–464.5)	348.3 (232.2–580.6)	< 0.01	464.5 (232.3–580.6)	464.5 (241.1–580.6)	0.06
Tea	241.1 (116.1–464.5)	232.2 (89.3–464.5)	0.04	160.8 (44.6–348.4)	160.8 (44.6–348.4)	0.87
Eggs	7.2 (4.5–17.9)	7.2 (4.5–17.9)	0.30	7.2 (4.5–17.9)	8.9 (7.2–17.9)	0.03
Potatoes	49.9 (24.9–89.7)	49.8 (29.9–89.7)	0.55	49.8 (29.9–89.7)	74.7 (44.9–89.7)	0.04
Refined grains	65.0 (48.6–93.1)	69.4 (46.2–102.4)	0.41	62.7 (42.0–97.8)	63.7 (41.3–95.6)	0.83
White, unprocessed meat	8.8 (5.2–13.6)	9.6 (5.4–13.9)	0.18	10.8 (6.3–15.2)	9.6 (6.3–14.5)	0.37
Cheese	21.4 (11.4–38.3)	23.8 (13.0–40.6)	0.27	26.1 (13.2–43.7)	24.9 (13.8–41.2)	0.41
Savory and ready products	90.1 (49.1–136.7)	82.9 (51.4–121.1)	0.44	76.0 (45.0–109.7)	79.4 (47.2–118.8)	0.07
Sugary products	71.3 (47.5–114.9)	77.5 (50.0–110.9)	0.53	68.3 (41.1–102.4)	69.7 (43.9–102.9)	0.24
Soup	22.3 (18.0–66.8)	35.8 (22.3–71.5)	0.04	35.8 (22.3–44.5)	35.8 (22.3–71.5)	< 0.01
Sweetened dairy	82.1 (44.4–147.7)	93.8 (46.5–143.0)	0.69	83.1 (33.7–136.0)	92.3 (44.9–141.1)	0.03
Artificially sweetened beverages	13.3 (0.0–111.8)	13.3 (0.0–80.3)	0.33	47.0 (0.0–143.4)	28.1 (0.0–129.1)	0.03
Sugar-sweetened beverages	120.1 (26.7–230.5)	88.8 (23.9–192.9)	0.03	66.3 (17.8–169.1)	75.3 (21.5–181.1)	0.48
Butter and hard margarines	16.1 (7.2–32.2)	19.2 (8.9–36.2)	0.03	20.1 (8.9–37.5)	21.5 (8.9–37.9)	0.54
Red and processed meat	58.1 (37.2–77.4)	61.8 (40.7–82.8)	0.03	63.0 (44.5–86.5)	68.1 (50.3–88.5)	0.02

Intake in median (IQR) in g/day. Between-group differences assessed by the Mann–Whitney *U* test

Cases in the overweight group had lower dietary intake of eggs (7.2 vs 8.9 g/day, p = 0.03), potatoes (49.8 vs 74.7 g/day, *p* = 0.04), soup (35.8 vs 35.8 g/day, *p* < 0.01), sweetened dairy (83.1 vs 92.3 g/day, *p* = 0.03), and red and processed meat (63.0 vs 68.1 g/day, *p* = 0.02). Furthermore, cases who were overweight consumed more artificially sweetened beverages (47.0 vs 28.1 g/day, *p* = 0.03) than controls. Other food groups were not statistically significantly different between the groups.

### Relative risk of food groups on incident adult-onset asthma

**Fig. 2:**
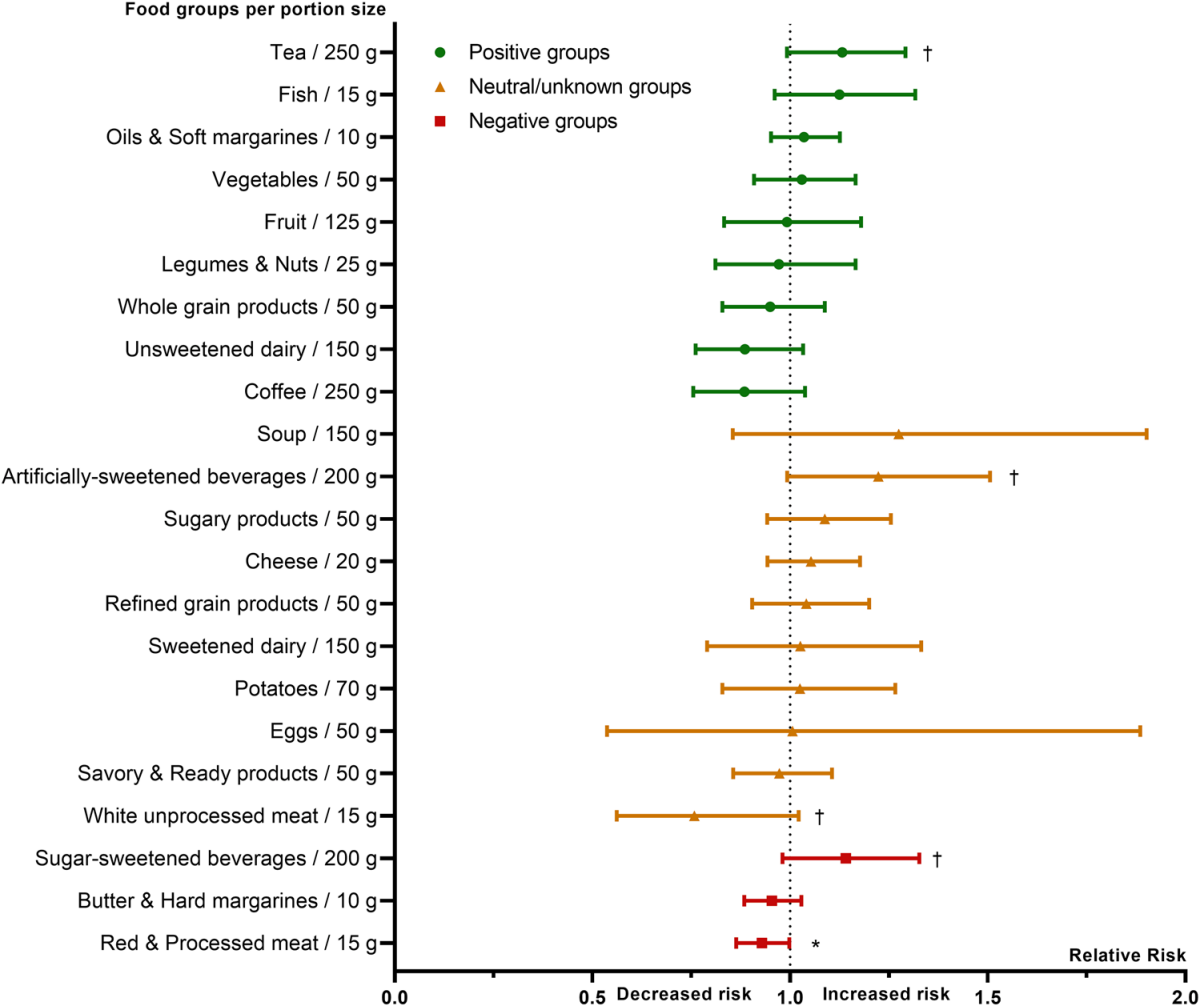
Relative risk of food groups on incident asthma in adults with BMI < 25 kg/m^2^. Log-binomial regression model, adjusted for sex, age, educational level and follow-up time. Relative risk (and 95% CI) is given per portion size of food groups. Dot: positive food groups; triangle: neutral/unknown food groups; square: negative food groups, according to Vinke et al. [42]. *p < 0.05; †*p* < 0.1

**Fig. 3:**
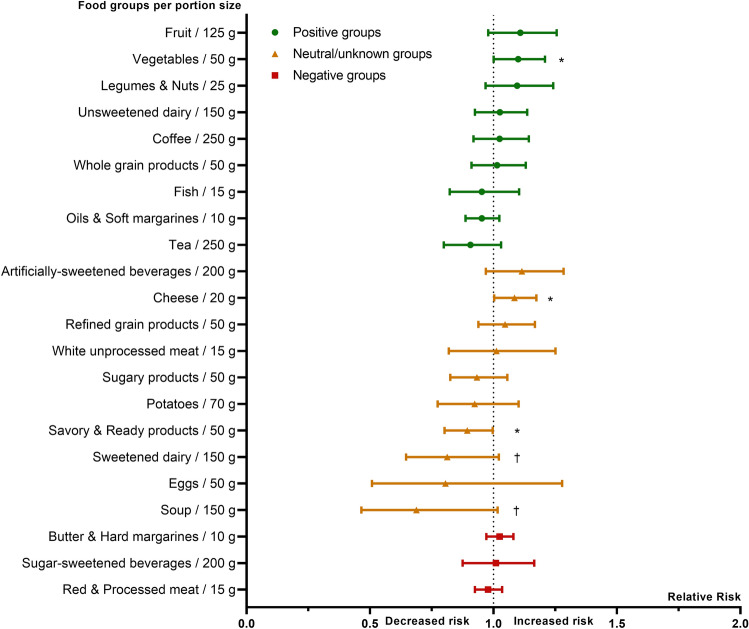
Relative risk of food groups on incident asthma in adults with BMI ≥ 25 kg/m^2^. Log-binomial regression model, adjusted for sex, age, educational level and follow-up time. Relative risk (and 95% CI) is given per portion size of food groups. Dot: positive food groups; triangle: neutral/unknown food groups; square: negative food groups, according to Vinke et al. [42]. *p < 0.05; †*p* < 0.1

To assess the risk of the various food groups on incident asthma in adults, a multivariable log-binomial regression analysis was performed, including the covariates sex, age, educational level and follow-up time. Results are shown in Fig. 2 for the normal weight group and in Fig. 3 for the overweight group.

In the normal weight group (Fig. 2), higher intake of 15 g of red and processed meat was statistically significant associated with a 7.1% lower risk of incident adult-onset asthma (RR 0.93 per 15 g; 95% CI 0.86–0.99; *p* = 0.04). Likewise, the higher intake of white unprocessed meat was borderline significantly associated with lower risk of adult-onset asthma (RR 0.76 per 15 g; 95% CI 0.56–1.02; *p* = 0.07). Furthermore, artificially sweetened beverages (RR: 1.22 per 200 g; 95% CI 0.99–1.51; *p* = 0.06), sugar-sweetened beverages (RR: 1.14 per 200 g; 95% CI 0.98–1.33; *p* = 0.09) and tea (RR: 1.13 per 250 g; 95% CI 0.99–1.29; *p* = 0.07) were borderline significantly linked to increased risk of incident asthma in normal weight adults.

In the group with a BMI ≥ 25 kg/m^2^ (Fig. 3), the risk of adult-onset asthma decreased with 10.6% for every 50 g higher intake of savory and ready products (RR: 0.89 per 50 g; 95% CI 0.80–0.99; *p* = 0.03). Higher consumption of cheese was associated with an increased risk of 8.5% of adult-onset asthma (RR: 1.09 per 20 g; 95% CI 1.00–1.17; *p* = 0.04) and vegetables with an increased risk of 10.0% (RR: 1.10 per 50 g; 95% CI 1.00–1.21; *p* = 0.05). Moreover, both higher intake of sweetened dairy (RR 0.81 per 150 g; 95% CI 0.65–1.02; *p* = 0.08) and soup (RR: 0.69 per 150 g; 95% CI 0.47–1.02; *p* = 0.06) were borderline significantly linked to lower risk of adult-onset asthma.

Overall, food groups labeled a priori as positive were not more often linked to a decreased risk, nor negative food groups to an increased risk of adult-onset asthma (Figs. 2 and 3). Additional adjustment for either food allergy or smoking, physical activity, alcohol use and energy intake affected significance levels, but not the magnitude or direction of the relative risks (see online supplement, table S2).

## Discussion

In a large population-based cohort, we explored the relation of diet quality and food intake with incident adult-onset asthma in normal weight and overweight adults. Our results show that diet quality—assessed by both the LLDS and MDS—was not associated with incident adult-onset asthma in both the BMI groups. Furthermore, no tendency was observed between the risk of incident asthma and the intake of a priori assumed favorable and unfavorable food groups. Although the dietary intake of several food groups differed between cases and controls, after adjustment for confounders only few food groups remained associated with incident adult-onset asthma. Overall, our results question the role of food as ‘simple’ risk factor of incident asthma in adults.

Several studies have been conducted on the association between dietary intake and incident adult-onset asthma, of which few assessed modification by BMI [13–29]. Overall, these studies report heterogeneous results (see online supplement, table S3). Dietary pattern approaches show increased risk or no association between a western diet and adult-onset asthma [13–17, 47]. ‘Fruit and vegetables’ or ‘nuts and wine’ dietary patterns were also not related to adult-onset asthma, as shown in a French cohort [15]. This is supported by the data of Australian adults where indeed no association was found between the Mediterranean Diet Score and incident asthma [18]. Yet, better diet quality—reflected by a higher Alternate Healthy Eating Index 2010 score—has been linked to lower risk in this Australian cohort, while no association was found in an US population [18, 19]. Both studies did not assess subgroups of BMI. Our study largely confirms and extends their results by showing no association of diet quality and adherence to a Mediterranean-style diet with incident adult-onset asthma within specific BMI subgroups.

Regarding individual foods, we found a reduced risk of asthma with the intake of red and processed meat in normal weight adults. In other studies high processed meat intake was not associated with asthma incidence [20–22], but was related to a higher asthma symptom score, with the effect being only partly mediated by BMI [48, 49]. More in line to previous findings, we observed a moderate effect of increased intake of sugar-sweetened beverages and artificially sweetened beverages on higher risk of adult-onset asthma in normal weight subjects, albeit only borderline significant. Also non-obese US adults consuming sweetened beverages ≥ 2 times/day, were more likely to have asthma than non-consumers [50]. In the Framingham Offspring Cohort, a strong effect of these beverages and fruit drinks—but not diet soda—on higher risk of incident asthma in adults was shown as well [29], which was attributed to the high fructose:glucose ratio in these drinks. However, our results do not fully support this hypothesis as we also found an increased risk of asthma for higher intake of artificially sweetened beverages.

In the overweight group, we unexpectedly observed a decreased risk of asthma for the intake of savory and ready products and an increased risk for higher cheese and vegetable consumption. No longitudinal study has yet examined the intake of savory and ready products on adult-onset asthma, but one study in German adults prospectively assessed the risk of cheese intake on incident asthma, which observed no association [22]. Furthermore, fruit and vegetables have been proposed to be protective for asthma development in two meta-analyses [51, 52], but again few longitudinal studies have been performed in adults. These two Scandinavian studies show decreased risk of incident asthma for higher consumption of flavonoid-rich fruits and by a combined fruit and fish consumption score [26, 28]. However, in line with our finding, in a large survey within the Global Allergy and Asthma European Network (GA^2^LEN), the intake of fruity vegetables was also positively associated with a higher asthma symptom score in European adults [53]. Interestingly, this association was most evident in the Dutch study center, which raises the question whether this can be attributed to the way vegetables are prepared in the Netherlands, as this may differ from habits in other countries. Indeed, 70% of Dutch people cook their vegetables by boiling, resulting in high loss of phytochemicals and consequently the nutritional quality of vegetables [54]. The general conclusion from the authors of the GA^2^LEN study was that there is no consistent evidence for an association of asthma with fruit and vegetable intake [53].

Given the robustness of our results and the lack of (consistent) findings in previous studies, we might start to question the straightforward role of diet in the adult-onset of such a multifactorial disease as asthma. Focusing solely on dietary factors—be it dietary patterns, foods or nutrients—might be too simple to elucidate the complex relationship of a Western lifestyle with adult-onset asthma, and an integrated approach might be necessary that assesses lifestyle patterns. Besides diet, physical activity and body composition as ‘classic’ lifestyle factors [30], this could also include smoking, drug use, socio-economic status and perhaps even stress or degree of urbanization. Although the current trend in studying diet-disease relationships is to assess dietary patterns and specific diet scores [30], most observational studies— including ours by using the MDS and LLDS—have shown no effects on incident adult-onset asthma. By using this approach, important signals may be missed due to competing risks of food components. For example, foods that are rich in omega-3 fatty acids or antioxidants may also contain histamine or pesticide residues, which could potentially be harmful for some distinct asthma phenotypes. Indeed, asthma is also no longer considered a single disease, but rather an umbrella diagnosis for several diseases with various clinical appearances [55]. These phenotypes vary according to the age of onset, atopic status, type of airway inflammation, and the association with obesity. Given this heterogeneity, better phenotyping is essential to understand the role of diet in the development and control of specific subtypes of asthma [31].

The strengths of our study are the prospective design of the study and the large sample size, which allowed well-powered stratified analyses. Furthermore, highly valuable dietary data was available from the externally validated Flower-FFQ, which is also representative for the Dutch population [37, 56]. In addition, the LLDS and food group categorization are in line with current scientific evidence on diet-disease relationships and reflect the adherence to the Dutch Dietary Guidelines [42]. The LLDS is a relatively new score that corresponds to the well-established MDS as both scores include vegetables, legumes, fruit and nuts as positive foods. Although the LLDS has not been validated against other well-known dietary indices—such as the Alternate Healthy Eating Index—it has the ability to discriminate between groups based on socio-demographic characteristics and has been related to other disease outcomes, such as chronic kidney disease and Crohn’s disease in previous studies [42, 57, 58]. Regarding the MDS, our Dutch study population may not have been representative enough for a traditional Mediterranean diet due to Westernized eating habits.

We next further confirmed the lack of association between diet and incident adult-onset asthma from a methodological point of view. First—as previously argued—combinations of foods and nutrients are consumed as meals rather than individual foods, which may interact with each other [30, 31]. Therefore, a sensitivity analysis was performed with a model containing all 22 food groups. Similar effect estimates were observed, although significance could not be reached due to the high number of parameters in the model (see online supplement, table S2). This excludes a confounding effect of overall eating pattern.

Second, since the follow-up time of our study was limited, incident asthma cases may have already improved their dietary intake at the start of the study due to asthma-related complaints, potentially leading to reverse causation. This could also explain the lower energy intake in overweight asthma cases compared to overweight controls. Perhaps, asthma cases with overweight might have followed an energy-restricted diet more often to lose weight in order to improve their asthma symptoms. To address reverse causation, the dietary intake data of asthma cases who reported asthma symptoms at baseline (10%) were compared to those cases without symptoms at baseline. The comparison gave no indication to consider reverse causation as explanation for our study findings.

Third, the applied asthma definition was based on self-reported new-onset asthma rather than physician-diagnosed asthma. Although the applied asthma definition is a common way to select new asthma cases in large cohort studies, this might explain the lack findings in our study. Due to privacy regulations, it was not possible to validate disease status with medical records. However, 64% of incident asthma cases reported asthma medication use at follow-up, which is often only prescribed by a medical doctor. In addition, cases showed increased lung function decline from baseline to follow-up, as compared to controls, showing that cases indeed developed respiratory problems over time. Furthermore, strict criteria were applied to select a population-at-risk free of airway disease at baseline, but selection bias was judged unlikely after comparison of demographic characteristics of the selected population-at-risk and total adult Lifelines population (see online supplement, table S1).

Fourth, missing important confounders may influence the outcomes. Therefore, a sensitivity analysis was performed to adjust for some other well-known confounders, which did not affect our results (see online supplement, table S2). Despite, residual confounding remains possible by other unknown factors.

Finally, the effect of dietary intake on incident adult-onset asthma could be mediated by BMI, due to for instance the inflammatory effect of fat mass and adipokines on asthma onset [30]. Although BMI is often considered a confounder in studies assessing diet-disease relationships, adjusting for a mediator might also lead to biased results [30]. Since BMI was significantly different between cases and controls in the overweight group, a sensitivity analysis was performed with inclusion of BMI as covariate, which showed similar results (see online supplement, table S2). Hence, both confounding and mediation by BMI seemed unlikely, although the latter was not assessed formally.

In summary, the results of this study do not provide convincing evidence for a role of diet quality and food intake in the etiology of adult-onset asthma. However, this does not preclude diet as risk factor for asthma in other time windows across the life-span or as disease modifier in asthma patients. Yet, to elucidate the complex relationship between a Western lifestyle and the risk of adult-onset asthma an integrative approach might be necessary, including a range of modifiable lifestyle factors. This also includes appropriate asthma phenotyping to account for the heterogeneity of this common disease.

## Supplementary Information

Below is the link to the electronic supplementary material.

Supplementary file1 (DOCX 48 KB)

## Data Availability

The manuscript is based on the data from the Lifelines cohort study. Lifelines adheres to standards for data availability. The data catalogue of the Lifelines cohort study is publicly accessible at www.lifelines.nl. All international researchers can obtain data at the Lifelines research office (research@lifelines.nl), for which a fee is required. The Lifelines research system allows access for reproducibility of the study results.

## Abbreviations

BMI: Body mass index
FEV_1_: Forced expiratory volume in 1 s
FFQ: Food frequency questionnaire
GA^2^LEN: Global Allergy and Asthma European Network
IQR: Interquartile range
LLDS: Lifelines diet score
MDS: Mediterranean diet score
MVPA: Moderate-to-vigorous physical activity
WHO: World Health Organization

## References

[1] de Nijs SB, Venekamp LN, Bel EH (2013). Adult-onset asthma: is it really different?. Eur Respir Rev.

[2] Global Initiative for Asthma (2022) Global Strategy for Asthma Management and Prevention. https://ginasthma.org/

[3] Amelink M, de Groot JC, de Nijs SB (2013). Severe adult-onset asthma: a distinct phenotype. J Allergy Clin Immunol.

[4] Aarab R, Vijverberg SJH, Prins M (2019). Prevalence of and factors associated with adult-onset asthma in different ethnic groups: the HELIUS study. Respir Med.

[5] Antó JM, Sunyer J, Basagaña X (2010). Risk factors of new-onset asthma in adults: a population-based international cohort study. Allergy.

[6] Räisänen P, Backman H, Hedman L (2021). High but stable incidence of adult-onset asthma in northern Sweden over the last decades. ERJ Open Res.

[7] Eagan TML, Brøgger JC, Eide GE, Bakke PS (2005). The incidence of adult asthma: a review. Int J Tuberc Lung Dis.

[8] Devereux G (2006). The increase in the prevalence of asthma and allergy: food for thought. Nat Rev Immunol.

[9] Dharmage SC, Perret JL, Custovic A (2019). Epidemiology of asthma in children and adults. Front Pediatr.

[10] Guilleminault L, Williams EJ, Scott HA (2017). Diet and asthma: is it time to adapt our message?. Nutrients.

[11] Alwarith J, Kahleova H, Crosby L (2020). The role of nutrition in asthma prevention and treatment. Nutr Rev.

[12] Kim J-H, Ellwood PE, Asher MI (2009). Diet and asthma: looking back, moving forward. Respir Res.

[13] Bédard A, Garcia-Aymerich J, Sanchez M (2015). Confirmatory factor analysis compared with principal component analysis to derive dietary patterns: a longitudinal study in adult women. J Nutr.

[14] Butler LM, Koh W-P, Lee H-P (2006). Prospective study of dietary patterns and persistent cough with phlegm among Chinese Singaporeans. Am J Respir Crit Care Med.

[15] Varraso R, Kauffmann F, Leynaert B (2009). Dietary patterns and asthma in the E3N study. Eur Respir J.

[16] Varraso R, Fung TT, Hu FB (2007). Prospective study of dietary patterns and chronic obstructive pulmonary disease among US men. Thorax.

[17] Varraso R, Fung TT, Barr RG (2007). Prospective study of dietary patterns and chronic obstructive pulmonary disease among US women. Am J Clin Nutr.

[18] Hlaing-Hlaing H, Dolja-Gore X, Tavener M (2021). Diet quality and incident non-communicable disease in the 1946–1951 cohort of the Australian Longitudinal Study on Women’s Health. Int J Environ Res Public Health.

[19] Varraso R, Chiuve SE, Fung TT (2015). Alternate Healthy Eating Index 2010 and risk of chronic obstructive pulmonary disease among US women and men: prospective study. BMJ.

[20] Jiang R, Camargo CA, Varraso R (2008). Consumption of cured meats and prospective risk of chronic obstructive pulmonary disease in women. Am J Clin Nutr.

[21] Varraso R, Jiang R, Barr RG (2007). Prospective study of cured meats consumption and risk of chronic obstructive pulmonary disease in men. Am J Epidemiol.

[22] Nagel G, Linseisen J (2005). Dietary intake of fatty acids, antioxidants and selected food groups and asthma in adults. Eur J Clin Nutr.

[23] Varraso R, Barr RG, Willett WC (2015). Fish intake and risk of chronic obstructive pulmonary disease in 2 large US cohorts. Am J Clin Nutr.

[24] Li J, Xun P, Zamora D (2013). Intakes of long-chain omega-3 (n−3) PUFAs and fish in relation to incidence of asthma among American young adults: the CARDIA study. Am J Clin Nutr.

[25] Mai X-M, Langhammer A, Chen Y, Camargo CA (2013). Cod liver oil intake and incidence of asthma in Norwegian adults—the HUNT study. Thorax.

[26] Uddenfeldt M, Janson C, Lampa E (2010). High BMI is related to higher incidence of asthma, while a fish and fruit diet is related to a lower–: results from a long-term follow-up study of three age groups in Sweden. Respir Med.

[27] Troisi RJ, Willett WC, Weiss ST (1995). A prospective study of diet and adult-onset asthma. Am J Respir Crit Care Med.

[28] Knekt P, Kumpulainen J, Järvinen R (2002). Flavonoid intake and risk of chronic diseases. Am J Clin Nutr.

[29] DeChristopher LR, Tucker KL (2018). Excess free fructose, high-fructose corn syrup and adult asthma: the Framingham Offspring Cohort. Br J Nutr.

[30] Bédard A, Li Z, Ait-Hadad W (2021). The role of nutritional factors in asthma: challenges and opportunities for epidemiological research. Int J Environ Res Public Health.

[31] Varraso R, Camargo CA (2016). Diet and asthma: need to account for asthma type and level of prevention. Expert Rev Respir Med.

[32] Muc M, Mota-Pinto A, Padez C (2016). Association between obesity and asthma—epidemiology, pathophysiology and clinical profile. Nutr Res Rev.

[33] Stolk RP, Rosmalen JGM, Postma DS (2008). Universal risk factors for multifactorial diseases. Eur J Epidemiol.

[34] Sijtsma A, Rienks J, der Harst P (2022). Cohort profile update: Lifelines, a three-generation cohort study and biobank. Int J Epidemiol.

[35] Chung KF, Wenzel SE, Brozek JL (2014). International ERS/ATS guidelines on definition, evaluation and treatment of severe asthma. Eur Respir J.

[36] World Health Organization (2000). Obesity: preventing and managing the global epidemic. Report of a WHO consultation. World Health Organ Tech Rep Ser.

[37] Brouwer-Brolsma EM, Perenboom C, Sluik D (2022). Development and external validation of the ‘Flower-FFQ’: a FFQ designed for the Lifelines cohort study. Public Health Nutr.

[38] National Institute for Public Health and the Environment (2019). Dutch food composition table 2019.

[39] Schofield WN (1985). Predicting basal metabolic rate, new standards and review of previous work. Hum Nutr Clin Nutr.

[40] Black AE (2000). Critical evaluation of energy intake using the Goldberg cut-off for energy intake: basal metabolic rate. a practical guide to its calculation, use and limitations. Int J Obes.

[41] Kromhout D, Spaaij CJK, de Goede J, Weggemans RM (2016). The 2015 Dutch food-based dietary guidelines. Eur J Clin Nutr.

[42] Vinke PC, Corpeleijn E, Dekker LH (2018). Development of the food-based Lifelines Diet Score (LLDS) and its application in 129,369 Lifelines participants. Eur J Clin Nutr.

[43] National Institute for Public Health and the Environment (2020) Portie-online, version 2020/1.4

[44] Trichopoulou A, Costacou T, Bamia C, Trichopoulos D (2003). Adherence to a mediterranean diet and survival in a Greek population. N Engl J Med.

[45] Wendel-Vos GCW, Schuit AJ, Saris WHM, Kromhout D (2003). Reproducibility and relative validity of the short questionnaire to assess health-enhancing physical activity. J Clin Epidemiol.

[46] Byambasukh O, Vinke P, Kromhout D (2021). Physical activity and 4-year changes in body weight in 52,498 non-obese people: the Lifelines cohort. Int J Behav Nutr Phys Act.

[47] Brigham EP, Kolahdooz F, Hansel N (2015). Association between Western diet pattern and adult asthma: a focused review. Ann Allergy Asthma Immunol.

[48] Andrianasolo RM, Hercberg S, Touvier M (2020). Association between processed meat intake and asthma symptoms in the French NutriNet-Santé cohort. Eur J Nutr.

[49] Li Z, Rava M, Bédard A (2017). Cured meat intake is associated with worsening asthma symptoms. Thorax.

[50] Park S, Akinbami LJ, McGuire LC, Blanck HM (2016). Association of sugar-sweetened beverage intake frequency and asthma among U.S. adults, 2013. Prev Med.

[51] Hosseini B, Berthon BS, Wark P (2017). Effects of fruit and vegetable consumption on risk of asthma, wheezing and immune responses: a systematic review and meta-analysis. Nutrients.

[52] Seyedrezazadeh E, Pour Moghaddam M, Ansarin K (2014). Fruit and vegetable intake and risk of wheezing and asthma: a systematic review and meta-analysis. Nutr Rev.

[53] Garcia-Larsen V, Arthur R, Potts JF (2017). Is fruit and vegetable intake associated with asthma or chronic rhino-sinusitis in European adults? Results from the Global Allergy and Asthma Network of Excellence (GA2 LEN) Survey. Clin Transl Allergy.

[54] Bongoni R, Verkerk R, Dekker M, Steenbekkers LPA (2015). Consumer behaviour towards vegetables: a study on domestic processing of broccoli and carrots by Dutch households. J Hum Nutr Diet.

[55] Kuruvilla ME, Lee FE-H, Lee GB (2019). Understanding asthma phenotypes, endotypes, and mechanisms of disease. Clin Rev Allergy Immunol.

[56] Baart AM, Brouwer-Brolsma EM, Perenboom CWM (2021). Dietary intake in the Lifelines cohort study: baseline results from the flower food frequency questionnaire among 59,982 participants. Nutrients.

[57] Peters V, Bolte L, Schuttert E (2022). Western and carnivorous dietary patterns are associated with greater likelihood of IBD development in a large prospective population-based cohort. J Crohns Colitis.

[58] Cai Q, Dekker LH, Vinke PC (2021). Diet quality and incident chronic kidney disease in the general population: the Lifelines cohort study. Clin Nutr.

