# Supplementary material for: Diet quality, food intake and incident adult-onset asthma: a Lifelines Cohort Study
Source: Eur J Nutr. 2023 Feb 4;62(4):1635–45. doi: 10.1007/s00394-023-03091-2 (PMC10195756; doi:10.1007/s00394-023-03091-2)
Supplement: Supplementary file 1 — Supplementary file1 (DOCX 48 KB) [file 394_2023_3091_MOESM1_ESM.docx]

**Supplementary information**

**Diet quality, food intake and incident adult-onset asthma: a Lifelines Cohort Study**

*European Journal of Nutrition*

Edith Visser^1,2^, Kim de Jong^1^, Janneke J.S. Pepels^1^, Huib A.M. Kerstjens^3^, Anneke ten Brinke^4^, Tim van Zutphen^2^

^1^ Department of Epidemiology, Medical Centre Leeuwarden, Leeuwarden, The Netherlands

^2^ Department of Sustainable Health, Faculty Campus Fryslân, University of Groningen, Leeuwarden, the Netherlands

^3^ Department of Pulmonary Medicine, University of Groningen, University Medical Centre Groningen, Groningen the Netherlands

^4^ Department of Pulmonary Medicine, Medical Centre Leeuwarden, Leeuwarden, The Netherlands

*Corresponding author:* Edith Visser – edith.visser@mcl.nl

**Supplementary information:**

**Table S1** Demographics of total adult Lifelines population, population-at-risk and excluded participants

**Table S2** Relative risk of food groups on incident adult-onset asthma in BMI groups, including sensitivity analyses

**Table S3** Overview of prospective studies on dietary factors and incident adult-onset asthma

**Table S1** Demographics of total adult Lifelines population, population-at-risk and excluded participants

|  |  | **Lifelines population ≥18y**  N=152,638 | **Population at-risk**  N=81,515 | **Excl. with  airway disease**  N=29,667 | **Excl. with  missing data**  N=41,456 |
| --- | --- | --- | --- | --- | --- |
| **Sex** | % female | 58.5 | 58.7 | 59.5 | 57.5 |
| **Age at inclusion** | years | 45.0 ± 13.1 | 44.5 ± 12.5 | 45.1 ± 13.2 | 46.0 ± 14.2 |
| **Educational level** | % low | 30.6 | 28.8 | 34.2 | 31.7 |
|  | % middle | 39.5 | 40.1 | 39.2 | 38.5 |
|  | % high | 29.8 | 31.1 | 26.5 | 29.7 |
| **BMI** | kg/m^2^ | 26.1 ± 4.3 | 26.0 ± 4.2 | 26.5 ± 4.7 | 26.0 ± 4.4 |
| **MVPA** | min/week | 260 (104-570) | 280 (120-640) | 260 (90-600) | 240 (90-460) |
| **Smoking status** | % current | 46.5 | 48.0 | 41.0 | 47.6 |
|  | % former | 21.3 | 19.7 | 26.6 | 20.7 |
|  | % never | 32.2 | 32.3 | 32.4 | 31.7 |
| **Smoking exposure ^b^** | pack-years | 8.8 (3.7-16.8) | 8.0 (3.5-15.0) | 11.0 (4.5-20.9) | 9.0 (3.8-17.0) |
| **Alcohol use** | % never | 20.3 | 21.0 | 21.7 | 18.0 |
|  | % low | 34.6 | 34.7 | 34.0 | 35.0 |
|  | % high | 45.0 | 44.4 | 44.3 | 47.0 |
| **Reliable FFQ data** | % yes | 89.8 | 86.4 | 89.6 | 96.8 |
| **Energy intake** **^c^** | kcal/day | 2064 ± 619 | 2071 ± 611 | 2059 ± 635 | 2053 ± 622 |
| **Food allergy** | % yes | 4.2 | 3.4 | 7.4 | 3.4 |
| **Nasal allergy** | % yes | 32.1 | 26.2 | 47.2 | 32.2 |
| **FEV_1_** | % predicted | 102.1 ± 14.1 | 104.5 ± 12.7 | 93.3 ± 15.0 | 103.1 ± 12.0 |
| **Eosinophils** | cells x10^9^/L | 0.15 (0.10-0.23) | 0.15 (0.10-0.22) | 0.18 (0.12-0.27) | 0.15 (0.10-0.23) |

BMI, body mass index; FEV_1_, forced expiratory volume in one second; MVPA, moderate-to-vigorous physical activity

^a^ Values are presented as %, mean ± SD or median (IQR)

^b^ Deviant sample size, in former and current smokers only

^c^ Deviant sample size, only reliable food data

**Table S2** Relative risk of food groups on incident adult-onset asthma in BMI groups, including sensitivity analyses

| **Food group** | **Portion size** | **Model** | **BMI <25 kg/m^2^** | | | |  | **BMI ≥25 kg/m^2^** | | | |
| --- | --- | --- | --- | --- | --- | --- | --- | --- | --- | --- | --- |
|  |  |  | **RR** | **LL** | **UL** | **P** |  | **RR** | **LL** | **UL** | **P** |
| Vegetables | 50 g  1 serving spoon | M1 | 1.03 | 0.91 | 1.17 | 0.64 |  | 1.10 | 1.00 | 1.21 | 0.05 |
|  |  | M2 | 1.04 | 0.91 | 1.18 | 0.60 |  | 1.12 | 1.01 | 1.24 | 0.03 |
|  |  | M3 | 1.03 | 0.91 | 1.17 | 0.63 |  | 1.10 | 1.00 | 1.21 | 0.05 |
|  |  | M4 | 0.95 | 0.79 | 1.15 | 0.61 |  | 1.10 | 0.97 | 1.25 | 0.15 |
|  |  | M5 | 1.02 | 0.90 | 1.16 | 0.71 |  | 1.10 | 1.00 | 1.21 | 0.06 |
| Fruit | 125 g  1 piece | M1 | 0.99 | 0.83 | 1.18 | 0.93 |  | 1.11 | 0.98 | 1.26 | 0.10 |
|  |  | M2 | 0.98 | 0.81 | 1.17 | 0.79 |  | 1.16 | 1.02 | 1.32 | 0.03 |
|  |  | M3 | 0.99 | 0.83 | 1.18 | 0.93 |  | 1.12 | 0.99 | 1.27 | 0.07 |
|  |  | M4 | 0.95 | 0.81 | 1.10 | 0.48 |  | 1.02 | 0.90 | 1.15 | 0.79 |
|  |  | M5 | 0.99 | 0.83 | 1.17 | 0.87 |  | 1.11 | 0.98 | 1.26 | 0.10 |
| Whole grain products | 50 g  1 serving spoon / 1 bread roll | M1 | 0.95 | 0.83 | 1.09 | 0.46 |  | 1.02 | 0.91 | 1.13 | 0.79 |
|  |  | M2 | 0.97 | 0.83 | 1.15 | 0.74 |  | 1.03 | 0.90 | 1.17 | 0.67 |
|  |  | M3 | 0.95 | 0.83 | 1.09 | 0.48 |  | 1.01 | 0.91 | 1.13 | 0.80 |
|  |  | M4 | 0.92 | 0.76 | 1.11 | 0.39 |  | 1.10 | 0.96 | 1.25 | 0.17 |
|  |  | M5 | 0.95 | 0.83 | 1.09 | 0.47 |  | 1.02 | 0.91 | 1.13 | 0.77 |
| Legumes & Nuts | 25 g  1 handful | M1 | 0.97 | 0.81 | 1.17 | 0.76 |  | 1.10 | 0.97 | 1.24 | 0.15 |
|  |  | M2 | 0.97 | 0.79 | 1.19 | 0.77 |  | 1.13 | 0.99 | 1.29 | 0.08 |
|  |  | M3 | 0.97 | 0.81 | 1.17 | 0.78 |  | 1.10 | 0.97 | 1.25 | 0.13 |
|  |  | M4 | 1.13 | 0.95 | 1.33 | 0.16 |  | 0.93 | 0.80 | 1.08 | 0.34 |
|  |  | M5 | 0.97 | 0.81 | 1.16 | 0.75 |  | 1.10 | 0.97 | 1.24 | 0.14 |
| Fish | 15 g  1 portion per week | M1 | 1.12 | 0.96 | 1.32 | 0.14 |  | 0.95 | 0.82 | 1.10 | 0.52 |
|  |  | M2 | 1.03 | 0.86 | 1.24 | 0.73 |  | 0.96 | 0.82 | 1.12 | 0.59 |
|  |  | M3 | 1.13 | 0.96 | 1.32 | 0.15 |  | 0.96 | 0.82 | 1.11 | 0.54 |
|  |  | M4 | 1.03 | 0.94 | 1.14 | 0.49 |  | 0.97 | 0.90 | 1.05 | 0.50 |
|  |  | M5 | 1.11 | 0.95 | 1.30 | 0.20 |  | 0.95 | 0.82 | 1.10 | 0.46 |
| Oils & Soft margarines | 10 g  1 table spoon | M1 | 1.04 | 0.95 | 1.13 | 0.42 |  | 0.95 | 0.89 | 1.02 | 0.19 |
|  |  | M2 | 1.05 | 0.96 | 1.16 | 0.28 |  | 0.93 | 0.86 | 1.01 | 0.09 |
|  |  | M3 | 1.04 | 0.95 | 1.13 | 0.42 |  | 0.95 | 0.89 | 1.02 | 0.16 |
|  |  | M4 | 0.90 | 0.77 | 1.05 | 0.17 |  | 1.01 | 0.91 | 1.12 | 0.80 |
|  |  | M5 | 1.04 | 0.95 | 1.13 | 0.39 |  | 0.96 | 0.89 | 1.03 | 0.21 |
| Unsweetened dairy | 150 g  1 small glass / dessert bowl | M1 | 0.89 | 0.76 | 1.03 | 0.12 |  | 1.03 | 0.93 | 1.14 | 0.63 |
|  |  | M2 | 0.86 | 0.72 | 1.01 | 0.07 |  | 1.01 | 0.90 | 1.14 | 0.82 |
|  |  | M3 | 0.89 | 0.76 | 1.03 | 0.12 |  | 1.03 | 0.93 | 1.14 | 0.62 |
|  |  | M4 | 0.90 | 0.77 | 1.05 | 0.17 |  | 1.01 | 0.91 | 1.12 | 0.80 |
|  |  | M5 | 0.90 | 0.77 | 1.04 | 0.15 |  | 1.03 | 0.93 | 1.15 | 0.52 |
| Coffee | 250 g  1 cup / mug | M1 | 0.88 | 0.75 | 1.04 | 0.13 |  | 1.03 | 0.92 | 1.14 | 0.66 |
|  |  | M2 | 0.86 | 0.72 | 1.02 | 0.09 |  | 1.03 | 0.91 | 1.16 | 0.62 |
|  |  | M3 | 0.88 | 0.75 | 1.03 | 0.12 |  | 1.03 | 0.92 | 1.14 | 0.64 |
|  |  | M4 | 0.95 | 0.80 | 1.13 | 0.54 |  | 0.99 | 0.88 | 1.12 | 0.93 |
|  |  | M5 | 0.89 | 0.76 | 1.05 | 0.16 |  | 1.03 | 0.93 | 1.15 | 0.58 |
| Tea | 250 g  1 cup / mug | M1 | 1.13 | 0.99 | 1.29 | 0.07 |  | 0.91 | 0.80 | 1.03 | 0.14 |
|  |  | M2 | 1.15 | 1.00 | 1.32 | 0.06 |  | 0.93 | 0.81 | 1.06 | 0.27 |
|  |  | M3 | 1.14 | 0.99 | 1.30 | 0.06 |  | 0.92 | 0.81 | 1.04 | 0.19 |
|  |  | M4 | 1.10 | 0.95 | 1.28 | 0.20 |  | 0.88 | 0.77 | 1.01 | 0.07 |
|  |  | M5 | 1.12 | 0.98 | 1.28 | 0.09 |  | 0.90 | 0.80 | 1.03 | 0.12 |
| Eggs | 50 g  1 egg | M1 | 1.01 | 0.54 | 1.89 | 0.98 |  | 0.81 | 0.51 | 1.28 | 0.36 |
|  |  | M2 | 0.99 | 0.51 | 1.93 | 0.98 |  | 0.84 | 0.52 | 1.35 | 0.46 |
|  |  | M3 | 1.00 | 0.53 | 1.87 | 1.00 |  | 0.78 | 0.49 | 1.23 | 0.29 |
|  |  | M4 | 0.95 | 0.88 | 1.03 | 0.23 |  | 0.98 | 0.92 | 1.05 | 0.58 |
|  |  | M5 | 1.00 | 0.53 | 1.87 | 1.00 |  | 0.79 | 0.50 | 1.26 | 0.32 |
| Potatoes | 70 g  1 serving spoon | M1 | 1.02 | 0.83 | 1.27 | 0.82 |  | 0.92 | 0.77 | 1.10 | 0.38 |
|  |  | M2 | 1.03 | 0.81 | 1.31 | 0.83 |  | 0.94 | 0.77 | 1.14 | 0.53 |
|  |  | M3 | 1.03 | 0.83 | 1.27 | 0.81 |  | 0.93 | 0.78 | 1.11 | 0.40 |
|  |  | M4 | 1.04 | 0.89 | 1.21 | 0.63 |  | 1.04 | 0.93 | 1.17 | 0.50 |
|  |  | M5 | 1.03 | 0.83 | 1.27 | 0.78 |  | 0.93 | 0.78 | 1.11 | 0.43 |

**Table S2** (continued)

| **Food group** | **Portion size** | **Model** | **BMI <25 kg/m^2^** | | | |  | **BMI ≥25 kg/m^2^** | | | |
| --- | --- | --- | --- | --- | --- | --- | --- | --- | --- | --- | --- |
|  |  |  | **RR** | **LL** | **UL** | **P** |  | **RR** | **LL** | **UL** | **P** |
| Refined grain products | 50 g  1 serving spoon / 1 bread roll | M1 | 1.04 | 0.90 | 1.20 | 0.58 |  | 1.05 | 0.94 | 1.17 | 0.41 |
|  |  | M2 | 0.95 | 0.80 | 1.13 | 0.54 |  | 1.05 | 0.93 | 1.19 | 0.45 |
|  |  | M3 | 1.04 | 0.90 | 1.20 | 0.57 |  | 1.05 | 0.94 | 1.17 | 0.40 |
|  |  | M4 | 0.81 | 0.59 | 1.12 | 0.20 |  | 1.01 | 0.81 | 1.26 | 0.90 |
|  |  | M5 | 1.04 | 0.90 | 1.20 | 0.59 |  | 1.05 | 0.94 | 1.17 | 0.42 |
| White unprocessed meat | 15 g  1 portion per week | M1 | 0.76 | 0.56 | 1.02 | 0.07 |  | 1.01 | 0.82 | 1.25 | 0.91 |
|  |  | M2 | 0.79 | 0.58 | 1.07 | 0.13 |  | 1.02 | 0.82 | 1.28 | 0.83 |
|  |  | M3 | 0.75 | 0.56 | 1.02 | 0.06 |  | 1.00 | 0.81 | 1.24 | 0.99 |
|  |  | M4 | 1.06 | 0.95 | 1.19 | 0.30 |  | 1.08 | 1.00 | 1.18 | 0.06 |
|  |  | M5 | 0.75 | 0.56 | 1.01 | 0.06 |  | 1.00 | 0.81 | 1.24 | 1.00 |
| Cheese | 20 g  1 portion for slice of bread | M1 | 1.05 | 0.94 | 1.18 | 0.36 |  | 1.09 | 1.00 | 1.17 | 0.04 |
|  |  | M2 | 1.08 | 0.96 | 1.22 | 0.21 |  | 1.13 | 1.04 | 1.22 | 0.01 |
|  |  | M3 | 1.05 | 0.94 | 1.18 | 0.35 |  | 1.09 | 1.00 | 1.18 | 0.04 |
|  |  | M4 | 0.94 | 0.82 | 1.08 | 0.37 |  | 0.89 | 0.79 | 1.00 | 0.05 |
|  |  | M5 | 1.05 | 0.94 | 1.17 | 0.36 |  | 1.08 | 1.00 | 1.17 | 0.04 |
| Savory & Ready products | 50 g  1 serving spoon | M1 | 0.97 | 0.86 | 1.11 | 0.68 |  | 0.89 | 0.80 | 1.00 | 0.04 |
|  |  | M2 | 0.95 | 0.82 | 1.10 | 0.51 |  | 0.83 | 0.72 | 0.94 | 0.00 |
|  |  | M3 | 0.97 | 0.85 | 1.11 | 0.66 |  | 0.89 | 0.79 | 0.99 | 0.03 |
|  |  | M4 | 1.11 | 0.95 | 1.30 | 0.21 |  | 0.97 | 0.85 | 1.12 | 0.71 |
|  |  | M5 | 0.97 | 0.86 | 1.11 | 0.70 |  | 0.89 | 0.80 | 1.00 | 0.04 |
| Sugary products | 50 g  1 large biscuit / cookie | M1 | 1.09 | 0.94 | 1.26 | 0.25 |  | 0.93 | 0.82 | 1.06 | 0.27 |
|  |  | M2 | 1.17 | 0.96 | 1.41 | 0.11 |  | 0.89 | 0.76 | 1.05 | 0.16 |
|  |  | M3 | 1.09 | 0.95 | 1.26 | 0.23 |  | 0.94 | 0.83 | 1.06 | 0.32 |
|  |  | M4 | 1.27 | 0.85 | 1.89 | 0.24 |  | 0.72 | 0.48 | 1.06 | 0.10 |
|  |  | M5 | 1.10 | 0.95 | 1.26 | 0.21 |  | 0.93 | 0.83 | 1.06 | 0.28 |
| Soup | 150 g  1 serving spoon | M1 | 1.28 | 0.85 | 1.90 | 0.23 |  | 0.69 | 0.47 | 1.02 | 0.06 |
|  |  | M2 | 1.30 | 0.85 | 1.99 | 0.23 |  | 0.70 | 0.46 | 1.06 | 0.09 |
|  |  | M3 | 1.28 | 0.85 | 1.90 | 0.23 |  | 0.68 | 0.46 | 1.00 | 0.05 |
|  |  | M4 | 0.98 | 0.75 | 1.28 | 0.87 |  | 0.83 | 0.66 | 1.05 | 0.13 |
|  |  | M5 | 1.27 | 0.85 | 1.89 | 0.24 |  | 0.69 | 0.46 | 1.01 | 0.06 |
| Sweetened dairy | 150 g  1 small glass / dessert bowl | M1 | 1.03 | 0.79 | 1.33 | 0.85 |  | 0.81 | 0.65 | 1.02 | 0.08 |
|  |  | M2 | 1.02 | 0.76 | 1.36 | 0.89 |  | 0.80 | 0.63 | 1.03 | 0.09 |
|  |  | M3 | 1.03 | 0.79 | 1.33 | 0.85 |  | 0.83 | 0.66 | 1.04 | 0.11 |
|  |  | M4 | 1.23 | 1.00 | 1.50 | 0.05 |  | 1.13 | 0.98 | 1.30 | 0.09 |
|  |  | M5 | 1.04 | 0.81 | 1.35 | 0.74 |  | 0.81 | 0.65 | 1.02 | 0.08 |
| Artificially sweetened beverages | 200 g  1 glass | M1 | 1.22 | 0.99 | 1.51 | 0.06 |  | 1.12 | 0.97 | 1.28 | 0.13 |
|  |  | M2 | 1.20 | 0.96 | 1.50 | 0.11 |  | 1.15 | 1.00 | 1.33 | 0.05 |
|  |  | M3 | 1.22 | 0.99 | 1.50 | 0.06 |  | 1.07 | 0.93 | 1.24 | 0.36 |
|  |  | M4 | 1.02 | 0.89 | 1.17 | 0.79 |  | 1.10 | 1.00 | 1.23 | 0.06 |
|  |  | M5 | 1.22 | 0.99 | 1.49 | 0.06 |  | 1.11 | 0.97 | 1.28 | 0.14 |
| Sugar-sweetened beverages | 200 g  1 glass | M1 | 1.14 | 0.98 | 1.33 | 0.09 |  | 1.01 | 0.88 | 1.16 | 0.89 |
|  |  | M2 | 1.19 | 1.00 | 1.41 | 0.05 |  | 1.00 | 0.85 | 1.17 | 0.96 |
|  |  | M3 | 1.14 | 0.98 | 1.33 | 0.08 |  | 1.00 | 0.87 | 1.16 | 0.98 |
|  |  | M4 | 0.96 | 0.82 | 1.12 | 0.62 |  | 1.00 | 0.90 | 1.10 | 0.98 |
|  |  | M5 | 1.15 | 0.99 | 1.33 | 0.08 |  | 1.00 | 0.87 | 1.16 | 0.95 |
| Butter & Hard margarines | 10 g  1 table spoon | M1 | 0.95 | 0.88 | 1.03 | 0.22 |  | 1.02 | 0.97 | 1.08 | 0.37 |
|  |  | M2 | 0.94 | 0.87 | 1.03 | 0.20 |  | 1.04 | 0.97 | 1.10 | 0.27 |
|  |  | M3 | 0.95 | 0.88 | 1.03 | 0.23 |  | 1.02 | 0.97 | 1.08 | 0.38 |
|  |  | M4 | 1.14 | 0.97 | 1.33 | 0.10 |  | 1.06 | 0.91 | 1.22 | 0.46 |
|  |  | M5 | 0.95 | 0.88 | 1.03 | 0.23 |  | 1.03 | 0.97 | 1.08 | 0.33 |
| Red & Processed meat | 15 g  1 slice of cold cuts / 1 portion per week | M1 | 0.93 | 0.86 | 1.00 | 0.04 |  | 0.98 | 0.93 | 1.03 | 0.44 |
|  |  | M2 | 0.93 | 0.86 | 1.00 | 0.06 |  | 0.97 | 0.91 | 1.03 | 0.36 |
|  |  | M3 | 0.93 | 0.86 | 1.00 | 0.04 |  | 0.97 | 0.92 | 1.03 | 0.30 |
|  |  | M4 | 0.96 | 0.87 | 1.05 | 0.33 |  | 1.04 | 0.97 | 1.11 | 0.26 |
|  |  | M5 | 0.93 | 0.87 | 1.00 | 0.05 |  | 0.98 | 0.93 | 1.04 | 0.49 |

BMI, body mass index; LL, lower limit 95% CI; LN, natural logarithm; RR, relative risk; UL, upper limit 95% CI

M1: sex, age, education, follow-up time + 1 food group (main model)

M2: M1 + alcohol use, pack years (LN), physical activity (LN), energy intake

M3: M1 + BMI

M4: M1 + 22 food groups

M5: M1 + food allergy

**Table S3** Overview of prospective studies on dietary factors and incident adult-onset asthma [13–29]

| **Ref.^a^** | **Author** | **Year** | **Country** | **Follow-up time** | **Asthma definition** | **Sample size^b^** | **Dietary assessment** | **Effect measure** | **Results^c,d^** |
| --- | --- | --- | --- | --- | --- | --- | --- | --- | --- |
| [13] | Bédard | 2015 | France | 12 y | SR. ‘Have you ever had an asthma attack’.  No at baseline – Yes at follow-up | 1,177 cases  29,412 controls  All female | FFQ. Dietary patterns by FA. | RR | ↑ Western diet  ↔ Prudent/healthy diet  ↔ Aperitif diet  No effect modification by BMI |
| [14] | Butler | 2006 | China | 5 y | SR. Age of asthma onset older than age at baseline. Asthma confirmed by symptoms and medication use in part of respondents. | 406 cases, 207 confirmed  45,104 controls | Quantitative FFQ. Dietary patterns by FA. | OR | ↔ Meat, sodium, carbs diet  ↔ Vegetable, fruit, soy diet |
| [15] | Varraso | 2009 | France | 10 y | SR. ‘Have you ever had an asthma attack, if so was this confirmed by a doctor’.  No at baseline – Yes at follow-up. | 628 cases  52,038 controls  All female | Dietary history questionnaire. Dietary patterns by FA. | RR | ↔ Western diet  ↔ Nuts and wine diet  ↔ Fruit, vegetable diet |
| [16] | Varraso | 2007 | USA | 12 y | SR. Doctor’s diagnosis of asthma and medication use in past 12 months.  No at baseline – Yes at follow-up. | 212 cases  42,705 controls  All male | Semi-quantitative FFQ. Dietary patterns by FA. | RR | ↔ Western diet  ↔ Prudent/healthy diet |
| [17] | Varraso | 2007 | USA | 16 y | SR. Doctor’s diagnosis of asthma and medication use in past 12 months.  No at baseline – Yes at follow-up. | 1,100 cases  70,943 controls  All female | Semi-quantitative FFQ. Dietary patterns by FA. | RR | ↔ Western diet  ↔ Prudent/healthy diet |
| [18] | Hlaing-Hlaing | 2021 | Australia | 15 y | SR. ‘have you been diagnosed or treated for asthma since the last survey’.  No at baseline – Yes at follow-up. | 374 cases  3,531 controls | Semi-quantitative FFQ. Diet quality indices were computed. | OR | ↓ Alternative Healthy Eating Index-2010  ↔ Mediterranean Diet Score  ↔ Healthy Eating Index for Australian Adult-2013 |
| [19] | Varraso | 2015 | USA | 16 y | SR. Doctor’s diagnosis of asthma and medication use in past 12 months.  No at baseline – Yes at follow-up. | 1,970 cases  118,284 controls | Semi-quantitative FFQ. Diet quality index was computed. | HR | ↔ Alternative Healthy Eating Index-2010 |
| [20] | Jiang | 2007 | USA | 16 y | SR. Doctor’s diagnosis of asthma.  No at baseline – Yes at follow-up. | 818 cases  70,713 controls  All female | Semi-quantitative FFQ | RR | ↔ Cured meat |
| [21] | Varraso | 2007 | USA | 12 y | SR. Doctor’s diagnosis of asthma and medication use in past 12 months.  No at baseline – Yes at follow-up. | 212 cases  42,705 controls  All male | Semi-quantitative FFQ | RR | ↔ Cured meat |
| [22] | Nagel | 2005 | Germany | 2 y | SR. Doctor’s diagnosis of asthma, additionally confirmed by questions on diagnostic tests, symptoms and medication use.  No at baseline – Yes at follow-up. | 105 cases  420 matched controls | Semi-quantitative FFQ | OR | ↑ Margarine  ↑ Oleic acid (C18:1)  ↔ Other fatty acids, including omega 3 and 6  ↔ Meat, cheese, milk, fish, eggs, butter, oils |
| [23] | Varraso | 2015 | USA | 16 y | SR. Doctor’s diagnosis of asthma and medication use in past 12 months.  No at baseline – Yes at follow-up. | 1,968 cases  118,207 controls | Semi-quantitative FFQ | HR | ↔ Fish  ↔ EPA & DHA |

**Tale S3** (continued)

| **Ref.^a^** | **Author** | **Year** | **Country** | **Follow-up time** | **Asthma definition** | **Sample size^b^** | **Dietary assessment** | **Effect measure** | **Results^c,d^** |
| --- | --- | --- | --- | --- | --- | --- | --- | --- | --- |
| [24] | Li | 2013 | USA | 20 y | SR. Doctor’s diagnosis of asthma or medication use.  No at baseline – Yes at follow-up. | 446 cases  3,716 controls | Interviewed quantitative FFQ | HR | ↓ Omega-3 fatty acids  ↓ EPA ↓ DHA  ↔ Non-fried fish  No effect modification by BMI |
| [25] | Mai | 2013 | Norway | 11 y | SR. ‘Do you have or have you had asthma?’  No at baseline – Yes at follow-up | 558 cases  16,970 controls | Questionnaire on cod liver oil consumption. | OR | ↑ Cod liver oil  No effect modification by BMI |
| [26] | Uddentfeldt | 2010 | Sweden | 13 y | SR. ‘Do you have or have you had asthma?’, ‘Have you ever been diagnosed with asthma by a doctor?’, ‘Do you use asthma medication?’ or ‘Do you have or have you had symptoms of asthma?’.  No at baseline – Yes at follow-up to any of the questions. | 791 cases  7,275 controls | 7 questions about consumption of fish, (game) meat, fruit, (fermented) milk, fast food. Consumption scores were computed. | RR | ↓ Fruit & fish consumption score  Other foods not reported |
| [27] | Troisi | 1995 | USA | 10 y | SR. Doctor’s diagnosis of asthma and medication use.  No at baseline – Yes at follow-up. | 760 cases  77,106 controls  All females | Semi-quantitative FFQ | RR | ↓ Tomato sauce  ↓ Vitamin E  ↑ Vitamin C with supplements  ↔ Vitamin A, Vitamin C, and carotene  ↔ (Saturated) fat, omega-3 fatty acids  ↔ Dark meat fish, tuna, shrimp  ↔ Nuts, peanut butter, breakfast cereals, cabbage, total fruit, apples, sweet potatoes, mushrooms, mayonnaise, oil/vinegar dressing |
| [28] | Knekt | 2002 | Finland | 28 y | By use of asthma medication from national register. Asthma free at baseline. | 382 cases  9,657 controls | Interviewed dietary history method | RR | ↓ Flavonoid intake  ↓ Apples  ↓ Oranges |
| [29] | DeChristopher | 2018 | USA | 17 y | SR. ‘Have you had asthma/wheeze since the last examination of short duration, long duration or with respiratory infections’.  No at baseline – Yes at follow-up. | 363 cases  2,333 controls | FFQ | HR | ↑ Non-diet soda, fruit drinks, apple juice  ↔ Diet soda, orange juice |

BMI, body mass index; DHA, docosahexaenoic acid; EPA, eicosapentaenoic acid; FA, factor analysis; FFQ, food frequency questionnaire; HR, hazard ratio; OR, odds ratio; RR, relative risk;
SR, self-reported

^a^ References can be found in the main article and are numbered accordingly

^b^ If number of controls was not explicitly mentioned, the number is derived from available data in the article

^c^ Only results of multivariable analyses

^d^ ↑ Increased risk, ↔ no association, or ↓ decreased risk of dietary factor on incident adult-onset asthma
